# Healthcare cost associations of patients who use illicit drugs in Florida: a retrospective analysis

**DOI:** 10.1186/s13011-020-00313-2

**Published:** 2020-09-29

**Authors:** Jessica L. Ryan, Veronica R. Rosa

**Affiliations:** University of West Florida, 11000 University Parkway, Pensacola, FL 32514 USA

**Keywords:** Illicit drug use, Healthcare costs, Charge-to-cost ratio, Opioids, Medicare, Medicaid

## Abstract

**Background:**

Illicit drug use increases visits to the hospital. Research is limited on the costs of these healthcare visits by illicit drug.

**Methods:**

Florida’s Agency for Health Care Administration’s emergency department and inpatient datasets from 2016 to 2018 were analyzed. Adults who used an illicit drug were included in the study population resulting in 709,658 observations. Cost-to-charge ratios were used to estimate healthcare costs. Linear regression analyzed associations of illicit drugs with total healthcare cost.

**Results:**

Total healthcare costs are estimated at $6.4 billion over the 3 year period. Medicare paid for the most patient care ($2.16 billion) with Medicaid and commercial insurance each estimated at $1.36 billion. Cocaine (9.25%) and multiple drug use (6.12%) increased the costs of an ED visit compared to a patient with cannabis SUD. Opioids (23.40%) and inhalants use (16.30%) increased the costs of inpatient compared to cannabis SUD.

**Conclusion:**

Healthcare costs are high of patients with illicit drug SUD and poisoning, over half of which are paid for with tax payer dollars and to an unknown degree hospital write-offs. Injuries and illness of patients using cocaine and multiple drugs are associated with more expensive ED patient care and opioids and inhalants are associated with more expensive inpatient care.

## Introduction

Nearly half of people have used an illicit drug at least once in their lifetime (National Institute of Drug Abuse [18]. In 2006, opioid-related fatalities were one of the highest causes of injury or death [27]. Between 2006 and 2011, the rates of those diagnosed with opioid addiction nearly tripled [12]. Further, Substance Abuse and Mental Health Services Administration (SAMHSA) found that 56.8 million people were diagnosed with either a substance use disorder (SUD) or mental health condition in 2017 [26]. Together, mental health and SUD had the highest disease burden in the U.S. in 2015 [11].

As substance use has increased, so has misuse and related events [27]. Consequently, there is an increase in healthcare services among individuals who have SUD [5, 7, 8, 20]. Individuals who are diagnosed with SUD are 2.2 times more likely to be hospitalized than those who do not misuse substances [8]. Specifically, there is an increase in utilization of acute care and corresponding high cost of care in the years before and after incidence of overdose [14]. Additionally, SUD can lead to indirect burdens on the healthcare system through long term effects of substance use [10].

In the U.S., rates and costs of inpatient stays associated with heroin and opioid overdose grew substantially from 2001 to 2012 [9]. U.S. annual societal costs are estimated at $740 billion in overall healthcare spending as well as lost productivity and crime due to all SUD [19]. According to The National Center for Injury Prevention and Control, the misuse of prescription drugs alone costs the nation at least $78.5 billion per year [6]. Substance misuse cost an estimated $1176 per capita and the full lifetime cost of illicit drug use was estimated at $43.9 billion in California in 2010 [16]. Hospital costs have been estimated at $2783 (in 2013 dollars) annually per person with a SUD involving illicit substances [8]. It is shown that Medicare recipients with a history of SUD had a mean 6 year cost as much as $56,178 more than those without history of SUD [5]. In 2007, Medicare and Medicaid were modified to include reimbursement opportunities to pay for screening and brief intervention for individuals at risk for substance misuse [24].

Medicaid funds more than one third of all substance use disorder treatment in the U.S. [4]. Moving forward, it is expected that expansions to the Patient Protection and Affordable Care Act (ACA) will increase healthcare spending among mental health and substance use disorder services [5]. Substance use prevalence significantly increased among Florida Medicaid recipients and more than tripled among the privately insured between 1999 and 2006 [27]. There is evidence that Medicaid enrollment increases while commercial plan enrollment decreases, following initial overdose for those with SUD [14].

Cocaine, prescription opioids, and heroin were found to be most commonly associated with overdoses [15]. White et al. [27] found that average annual per patient cost was greater for patients specifically using opioids over other substances. Recently, there is a growing trend in synthetic opioids related to overdose deaths [22]. Another study indicated sedatives account for the most intentional overdoses, followed by benzodiazepines such as Xanax and Valium [21]. More than 1 in 4 people using heroin can be expected to have a hospital admission every year [8]. Further research is needed on healthcare use and spending in different SUD user groups [2, 8]. In addition, there is currently not enough standardized cost information for policy makers and funding agencies to use for allocation of support to substance use disorder treatment programs and services [1, 26]. Some efforts, such as the Florida Brief Intervention and Treatment for Elders (BRITE) project and Screening Brief Intervention and Referral to Treatment (SBIRT), aim to screen and serve individuals at risk for misusing substances [24]. Ideally, these services and referral opportunities may be expanded, updated, and utilized by a wider population. As of March 2020, the National Institute on Drug Abuse (NIDA) was citing a 2007 study as the latest available national cost estimates for illicit drug use [19].

Addiction to illicit drugs can be treated and overdoses and injury prevented [23]. However, this requires not only identifying patients with SUD, but determining the subgroups to target – of particular interest is which populations have the highest healthcare costs. The first objective of this research is to identify the healthcare costs of emergency department (ED) patients and inpatients with illicit drug SUD or overdose in Florida. The second objective of this research is to analyze the associations of healthcare costs of patients with illicit drug SUD or overdose in Florida with demographics, payer, drug type, physical disorders common with SUD, and mental health disorders. The overarching goal of this research is to help researchers further understand the differences in illicit drug use with healthcare costs and to inform policy and decision makers for targeted treatment and allocation of scarce resources.

## Methods

The Florida Agency for Health Care Administration’s (AHCA’s) ED, inpatient, and financial datasets were used for this research. All datasets are de-identified and available to the public. The ED and inpatient dataset includes demographics, diagnosis codes, length of stay (LOS), and charges. The financial dataset includes each Florida acute care hospital’s ownership status as well as total revenues and total expenses. The study population included all ED and inpatients, aged 18 and over, in Florida from 2016 to 2018 who had a principal or other diagnosis code that indicated illicit substance use. International Statistical Classification of Diseases and Related Health Problems Clinical Modification (ICD-10-CM) codes were used to identify patients having a SUD or poisoning (overdose) based on a chart of ICD-10-CM codes of drug abuse by the California Department of Health [3]. The illicit drug categories generally followed ICD-10-CM categories; for example, patients with codes beginning with F12 ‘cannabis related disorders’ or T40 codes related to cannabis poisoning were coded as cannabis users. The drug categories from ICD-10-CM included opioids, cannabis, sedatives, cocaine, stimulants, hallucinogens, and inhalants. If a patient’s record indicated they had more than one illicit drug SUD during their visit, they were categorized as using multiple drugs. The initial count for ED observations was 355,758 but 40,614 were missing financial data. The initial count for inpatient observations was 515,559 but 120,720 were missing financial information. The final study population included 709,658 patients: 314,727 ED and 394,841 inpatients. SAS 9.4 and Microsoft Excel were used to analyze the data.

Descriptive statistics as well as two linear regression models were analyzed. The regression models both had total cost as the dependent variable and were analyzed with the ED and inpatient datasets separately. Independent variables of the models included demographics, payer, LOS, trauma alert status, physical and mental health comorbidities commonly associated with SUD, drug groups, and hospital factors such as teaching and ownership status. Mental health disorders were controlled for because they have been found to increase the risk of relapse and total healthcare spending for those with SUD [4, 13].

Demographics used in the analysis were included in the datasets. Rural designation was based on patient county and the Florida Department of Health’s rural classification of counties. Physical and mental comorbidities of the patients were coded based on an ICD-10-CM diagnosis code listing from the Minnesota Department of Human Services [17] and common comorbidities of people with SUD according to SAMHSA [25]. Marijuana was not found to be associated with inpatient stays while other illicit drug SUD were [8]. Therefore, marijuana was used as the reference group in both regression models.

Total healthcare costs were estimated from reported charges by using total hospital cost-to-charge ratios. This method was based off prior research by Hsu et al. [9]. The cost-to-charge ratios were total patient care services expense divided by total patient care services revenue for each hospital for each year as reported in the AHCA financial datasets. Costs for 2016 and 2017 were then adjusted to 2018 dollars using the hospital producer price index as reported by the Bureau of Labor Statistics. As healthcare charges and costs can skew heavily, the total costs in 2018 dollars for each patient were then log transformed. The total costs in 2018 dollars were used in the descriptive statistics, while the log transformed total cost in 2018 dollars was the dependent variable in the regression models. To interpret the results of the log transformed cost regression models, the formula “1 minus exponential to the x” is used to convert the estimates back into real units.

## Results

Demographics by illicit drug type are provided in Table 1 for ED and inpatient visits combined. There were more female inpatients than males for opioids and sedatives use. Hispanic patients had a range of illicit drug use percentages from 7.9% (stimulants) to 14.5% (hallucinogens). Black patients had a range of illicit drug use percentages from 6.0% (sedatives) to 45.2% (cocaine). Patients of other races ranged from 4.7% (opioids, sedatives, and multiple drugs) to 10.4% (inhalants). White patients with illicit drug use ranged from 50% (cocaine) to 89.3% (sedatives). Medicare paid for 38.7% of all patient visits with SUD of opioids and nearly the same for SUD of sedatives (35.67) while only 7.3% of stays with SUD of hallucinogens. Medicaid inpatients with SUD ranged from 16.0% (sedatives) to 28.8% (cocaine). All patient SUD groups had a trauma alert percentage below 1%, except for inhalants (3.2%). Patient visits associated with illicit drug use increased from 2016 to 2018 in every group, except for opioids and inhalants. The total counts of patients by drug group varied greatly from 1406 (inhalants) to 267,067 (opioids).

**Table 1:** Demographics of ED and Inpatients by Illicit Drug Type in Florida, 2016–2018

		Opioids	Sedatives	Cocaine	Stimulants	Hallucinogens	Inhalants	Multiple Drugs	Cannabis	Total Counts
**Gender**	Female	52.0%	54.8%	33.0%	40.5%	24.2%	31.0%	38.7%	38.0%	304,815
	Male	48.0%	45.2%	67.0%	59.5%	75.8%	69.0%	61.3%	62.0%	405,170
**Ethnicity**	Hispanic	8.5%	12.0%	13.3%	7.9%	14.5%	10.9%	11.1%	11.5%	74,192
	Non-Hispanic	91.5%	88.0%	86.7%	92.1%	85.5%	89.1%	88.9%	88.5%	635,793
**Race**	Black	10.5%	6.0%	45.2%	10.3%	23.1%	20.8%	22.1%	34.1%	164,808
	Other Race	4.7%	4.7%	4.8%	5.1%	8.6%	10.4%	4.7%	5.8%	35,236
	White	84.8%	89.3%	50.0%	84.6%	68.2%	68.8%	73.2%	60.2%	509,941
**Payer**	Uninsured	23.7%	22.8%	37.3%	47.7%	45.1%	37.4%	41.6%	35.3%	232,718
	Medicare	38.7%	35.7%	18.9%	12.6%	7.3%	14.7%	16.0%	13.7%	177,106
	Medicaid	17.6%	16.0%	28.8%	20.4%	16.9%	20.0%	24.0%	26.1%	159,269
	Other Insurance	3.9%	4.0%	5.7%	5.6%	5.4%	8.4%	5.7%	5.5%	34,809
	Commercial	16.2%	21.5%	9.3%	13.8%	25.3%	19.5%	12.8%	19.5%	106,083
**Trauma Alert**	Trauma Alert	0.4%	0.7%	0.8%	0.8%	0.9%	3.2%	0.7%	0.7%	4324
	Non Trauma Alert	99.6%	99.3%	99.2%	99.2%	99.1%	96.8%	99.3%	99.3%	705,661
**Year**	2016	32.6%	29.4%	30.8%	26.1%	28.6%	45.2%	30.2%	31.2%	221,427
	2017	34.4%	31.7%	34.0%	31.1%	31.1%	33.5%	33.2%	33.7%	239,691
	2018	32.9%	38.9%	35.2%	42.8%	40.4%	21.3%	36.6%	35.1%	248,867
**Average**	Age (STD)	49.5 (23.4)	49.6 (19.3)	45.2 (16.0)	38.5 (22.7)	30.5 (24.8)	40.6 (13.9)	39.6 (19.0)	37.0 (18.4)	
	LOS (inpatients) (STD)	6.42 (9.9)	5.31 (7.5)	5.15 (9.5)	4.57 (9.1)	5.24 (14.9)	6.57 (10.5)	5.32 (9.5)	4.54 (6.8)	
	**Total Counts**	267,067	17,711	133,307	38,634	2037	1406	120,759	129,064	709,985

Healthcare costs from ED and inpatient visits associated with illicit drug use are reported in Table 2. Total costs are estimated at $6.4 billion over the three year period. Medicare paid for the most patient care ($2.16 billion) with Medicaid and commercial insurance both around $1.36 billion. Uninsured patients accounted for over $1 billion in healthcare costs. Patients with an opioid SUD had the greatest total costs at $2.98 billion from 2016 to 2018, followed by cocaine ($1.3 billion), multiple drug users ($965.2 million), cannabis ($764.0 million), stimulants ($218.6 million), sedatives ($123.1 million), inhalants ($11.3 million), and hallucinogens ($8.7 million). Healthcare costs of patients with cocaine SUD more than doubled from 2017 ($312.4 million) to 2018 ($726.8 million).

**Table 2:** Healthcare Costs of Patients by Illicit Drug in Florida, 2016–2018

Payer	Opioids	Sedatives	Cocaine	Stimulants	Hallucinogens	Inhalants	Multiple Drugs	Cannabis	Total Costs
Uninsured	$303,580,273	$12,651,381	$260,645,873	$77,488,376	$3,037,384	$2,090,506	$322,417,982	$206,172,674	$1,188,084,449
Medicare	$1,502,506,095	$60,431,863	$207,258,281	$41,906,209	$1,077,986	$2,328,874	$189,335,130	$156,375,886	$2,161,220,324
Medicaid	$514,680,922	$17,562,632	$308,104,203	$52,981,163	$1,996,175	$2,900,562	$271,772,453	$194,873,971	$1,364,872,081
Other Insurance	$128,885,460	$4,674,212	$60,814,707	$15,463,740	$677,578	$1,670,357	$60,377,110	$53,009,490	$325,572,654
Commercial	$534,096,623	$27,738,560	$492,611,678	$30,739,067	$1,904,929	$2,273,589	$121,246,188	$153,570,258	$1,364,180,892
**Year**									
2016	$984,401,523	$35,857,269	$290,192,067	$57,384,749	$2,390,132	$5,472,874	$294,398,297	$246,195,072	$1,916,291,983
2017	$991,568,122	$38,220,270	$312,437,040	$67,421,012	$2,945,091	$4,404,895	$319,423,204	$255,874,046	$1,992,293,680
2018	$1,007,779,728	$48,981,109	$726,805,635	$93,772,794	$3,358,829	$1,386,119	$351,327,362	$261,933,161	$2,495,344,737
**Total Costs**	$2,983,749,373	$123,058,648	$1,329,434,742	$218,578,555	$8,694,052	$11,263,888	$965,148,863	$764,002,279	$6,403,930,400

The results from the ED regression are reported in Table 3. This regression analyzed associations of patients with illicit drug use and their healthcare costs. The independent variables of interest were the illicit drug groups. Cocaine (9.25%) and multiple drug use (6.12%) increased the costs of an ED visit compared to a patient with cannabis SUD. Opioids (-30%), sedatives (-19.32%), stimulants (-1.84%), hallucinogens (-7.9%), and inhalants (-13.37%) all lowered the cost of ED care compared to cannabis. Patients with illicit drug use cost of care increased with Medicare (8.33%) and other insurance (8.25%) compared to ED patients with commercial insurance. Uninsured inpatients had lower cost of care by 9.33% and Medicaid inpatients had costs 1.85% lower.

**Table 3:** Regression Model of Healthcare Costs of ED Patients with Illicit Drug SUD

Column1	Variable	Parameter Estimate	Standard Error	Pr > |t|	% Change to Cost
	Intercept	6.8428	0.0062	<.0001	
Gender	**Female**^**a**^	**0.0554**	**0.0034**	**<.0001**	**5.69%**
	Male (ref.)	**–**	**–**	**–**	
Age	**Age**^**a**^	**0.0034**	**0.0001**	**<.0001**	**0.34%**
Ethnicity	**Hispanic**^**a**^	**0.0634**	**0.0056**	**<.0001**	**6.55%**
	Non-Hispanic (ref.)	**–**	**–**	**–**	
Race	Black	0.0046	0.0043	0.2773	
	**Other**^**a**^	**0.0376**	**0.0078**	**<.0001**	**3.83%**
	White (reference)	**–**	**–**	**–**	
Rural	**Rural**^**a**^	**0.0771**	**0.0085**	**<.0001**	**8.01%**
	Urban (ref.)	**–**	**–**	**–**	
Payer	**Uninsured**^**a**^	**−0.098**	**0.0049**	**<.0001**	**−9.33%**
	**Medicare**^**a**^	**0.08**	**0.0063**	**<.0001**	**8.33%**
	**Medicaid**^**a**^	**−0.0187**	**0.0055**	**0.0007**	**−1.85%**
	**Other**^**a**^	**0.0793**	**0.0087**	**<.0001**	**8.25%**
	Commercial (ref.)	**–**	**–**	**–**	
LOS	**LOS**^**a**^	**0.559**	**0.0023**	**<.0001**	**74.90%**
Trauma Alert	**Trauma Alert**^**a**^	**1.668**	**0.0398**	**<.0001**	**430.16%**
	Non-Trauma Alert (ref.)	**–**	**–**	**–**	
Physical Comorbidities	**Liver Disease**^**a**^	**0.3739**	**0.0161**	**<.0001**	**45.34%**
	**Severe Liver Disease**^**a**^	**0.4058**	**0.0970**	**<.0001**	**50.05%**
	HIV/AIDS	−0.0083	0.0193	0.6675	
	None (ref.)	–	–	–	
Mental Comorbidities	**Anxiety**^**a**^	**0.1225**	**0.0057**	**<.0001**	**13.03%**
	Mood	0.0455	0.0279	0.1024	
	**Schizophrenia and Personality**^**a**^	**−0.1118**	**0.0087**	**<.0001**	**−10.58%**
	**Bipolar**^**a**^	**0.0308**	**0.0083**	**0.0002**	**3.13%**
	**Depression**^**a**^	**0.0418**	**0.0067**	**<.0001**	**4.27%**
	Conduct	−0.0245	0.0463	0.5962	
	**PTSD**^**a**^	**−0.028**	**0.0137**	**0.0402**	**−2.77%**
	**ADHD**^**a**^	**−0.0807**	**0.0187**	**<.0001**	**−7.76%**
	None (ref.)	**–**	**–**	**–**	
Drugs	**Opioids**^**a**^	**−0.3567**	**0.0047**	**<.0001**	**−30.00%**
	**Sedatives**^**a**^	**−0.2146**	**0.0108**	**<.0001**	**−19.32%**
	**Cocaine**^**a**^	**0.0885**	**0.0051**	**<.0001**	**9.25%**
	**Stimulants**^**a**^	**−0.0186**	**0.0072**	**0.0096**	**−1.84%**
	**Hallucinogens**^**a**^	**−0.0823**	**0.0250**	**0.001**	**−7.90%**
	**Inhalants**^**a**^	**−0.1435**	**0.0316**	**<.0001**	**−13.37%**
	**Multiple Drugs**^**a**^	**0.0594**	**0.0058**	**<.0001**	**6.12%**
	Cannabis (ref.)	**–**	**–**	**–**	
Teaching Hospital	**Teaching**^**a**^	**−0.2618**	**0.0059**	**<.0001**	**−23.03%**
	Non-Teaching (ref.)	**–**	**–**	**–**	
Hospital Ownership	**For-Profit**^**a**^	**0.0830**	**0.0038**	**<.0001**	**8.65%**
	**Government**^**a**^	**−0.2420**	**0.0049**	**<.0001**	**−21.49%**
	Not-For-Profit (ref.)	**–**	**–**	**–**	

^a^statistically significant at the α = 0.05 level

The results from the inpatient regression are reported in Table 4. This regression was modeled the same as the ED regression. Opioids (23.40%) and inhalants (16.30%) increased the costs of inpatients compared to cannabis. Sedatives (-14.80%), cocaine (-6.87%), stimulants (-7.435), hallucinogens (-12.00), and using multiple drugs (-10.33%) all lowered healthcare costs compared to cannabis. In addition, having Medicare raised inpatient healthcare costs by 2.85% compared to patients with commercial insurance. Having Medicaid (-6.77%), being uninsured (-9.83%), or having other insurance (-5.15%) all lowered healthcare costs compared to commercial patients.

**Table 4:** Regression Model of Healthcare Costs of Inpatients with Illicit Drug SUD

	Variable	Parameter Estimate	Standard Error	Pr > |t|	% Change to Cost
	Intercept	8.3235	0.0059	<.0001	
Gender	Female	− 0.0005	0.0027	0.8552	
	Male (ref.)	–	–	–	
Age	**Age**^**a**^	**0.0067**	**0.0001**	**<.0001**	**0.68%**
Ethnicity	**Hispanic**^**a**^	**−0.0250**	**0.0046**	**<.0001**	**−2.47%**
	Non-Hispanic (ref.)	**–**	**–**	**–**	
Race	**Black**^**a**^	**−0.0394**	**0.0034**	**<.0001**	**−3.87%**
	**Other**^**a**^	**0.1217**	**0.0064**	**<.0001**	**12.94%**
	White (ref.)	–	–	–	
Rural	Rural	−0.0075	0.0070	0.2864	
	Urban (ref.)	**–**	**–**	**–**	
Payer	**Uninsured**^**a**^	**−0.1035**	**0.0044**	**<.0001**	**−9.83%**
	**Medicare**^**a**^	**0.0281**	**0.0043**	**<.0001**	**2.85%**
	**Medicaid**^**a**^	**−0.0701**	**0.0044**	**<.0001**	**−6.77%**
	**Other Insurance**^**a**^	**−0.0529**	**0.0067**	**<.0001**	**−5.15%**
	Commercial (ref.)	**–**	**–**	**–**	
LOS	**LOS**^**a**^	**0.0501**	**0.0001**	**<.0001**	**5.14%**
Trauma Alert	**Trauma Alert**^**a**^	**1.1036**	**0.0133**	**<.0001**	**201.51%**
	Non-Trauma Alert (ref.)	**–**	**–**	**–**	
Physical Comorbidities	**Liver Disease**^**a**^	**0.1868**	**0.0052**	**<.0001**	**20.54%**
	**Severe Liver Disease**^**a**^	**0.2642**	**0.0145**	**<.0001**	**30.23%**
	**HIV/AIDS**^**a**^	**0.1325**	**0.0108**	**<.0001**	**14.17%**
	None (ref.)	–	–	–	
Mental Comorbidities	Anxiety	−0.0023	0.0031	0.4604	
	**Mood**^**a**^	**−0.1366**	**0.0110**	**<.0001**	**−12.77%**
	**Schizophrenia and Personality**^**a**^	**−0.2184**	**0.0050**	**<.0001**	**−19.62%**
	**Bipolar**^**a**^	**0.0993**	**0.0050**	**<.0001**	**10.43%**
	**Depression**^**a**^	**0.0555**	**0.0035**	**<.0001**	**5.71%**
	**Conduct**^**a**^	**−0.4158**	**0.0286**	**<.0001**	**−34.02%**
	**PTSD**^**a**^	**−0.1844**	**0.0057**	**<.0001**	**−16.84%**
	**ADHD**^**a**^	**−0.1264**	**0.0102**	**<.0001**	**−11.87%**
	None (ref.)	**–**	**–**	**–**	
Drugs	**Opioids**^**a**^	**0.2103**	**0.0042**	**<.0001**	**23.40%**
	**Sedatives**^**a**^	**−0.1602**	**0.0091**	**<.0001**	**−14.80%**
	**Cocaine**^**a**^	**−0.0711**	**0.0047**	**<.0001**	**−6.87%**
	**Stimulants**^**a**^	**−0.0772**	**0.0072**	**<.0001**	**−7.43%**
	**Hallucinogens**^**a**^	**−0.1279**	**0.0313**	**<.0001**	**−12.00%**
	**Inhalants**^**a**^	**0.1510**	**0.0345**	**<.0001**	**16.30%**
	**Multiple Drugs**^**a**^	**−0.1090**	**0.0045**	**<.0001**	**−10.33%**
	Cannabis (ref.)	**–**	**–**	**–**	
Teaching Hospital	**Teaching**^**a**^	**0.1632**	**0.0045**	**<.0001**	**17.73%**
	Non-Teaching (ref.)	**–**	**–**	**–**	
Hospital Ownership	**For-Profit**^**a**^	**0.0149**	**0.0029**	**<.0001**	**14.96%**
	**Government**^**a**^	**−0.0957**	**0.0042**	**<.0001**	**−9.13%**
	Not-For-Profit (ref.)	**–**	**–**	**–**	

^a^statistically significant at the α = 0.05 level

## Discussion

There were fewer observations from the ED dataset than the inpatient dataset for patients with illicit drug use. With other disease and injury research, there are usually greater observations in the ED because a small percentage of those get admitted. Patients with illicit drug use who seek care from a hospital appear to have a high admittance rate, which implies their injuries/illnesses are severe. Counts and healthcare costs of patients with an illicit drug SUD or poisoning in their records increased each year in Florida from 2016 to 2018. Either drug use is increasing in Florida or the people who use illicit drugs are getting injured or sick at a higher rate. In total, illicit drug use cost $ 6,403,930,400 over 3 years of which over half ($3,526,092,405) was paid from public insurance. Uninsured patients cost $1,188,084,449 in patient visits; it is unknown from the dataset how much of this the hospitals will write off as charity care or bad debt. The total costs of healthcare associated with illicit drug use are much higher as this study did not include ambulatory care or rehabilitation costs. Although, it is clear illicit drugs cost the healthcare system a large amount of money each year. As policy makers have limited resources for the prevention and treatment of illicit drug use, the second objective of this research was to identify associations of illicit drugs with cost.

All illicit drug groups were found to be statistically associated with healthcare costs compared to cannabis. Interestingly though, only cocaine and multiple drugs were associated with higher ED costs and only opioids and inhalants were associated with higher inpatient costs. Sedatives, stimulants, and hallucinogens were all associated with lower healthcare costs than cannabis when demographics, physical and mental comorbidities, and hospital factors were controlled. In Florida, the opioid group had the most patients and the highest costs from 2016 to 2018, which is not surprising considering the current epidemic the country is experiencing. Inpatients who used inhalants were shown to have a greater percentage of trauma alerts and average longer LOS, which would indicate a greater severity of injury. This may explain why inpatient costs would be higher for this group.

There are limitations to this study. One, the data includes Florida hospitals and may not be indicative of the rest of the country. Secondly, cost estimates are used as the datasets only have charges reported. However, the estimates of healthcare costs should be conservative as over 160,000 observations were not included due to missing data. In addition, some SUD may not be reported during hospital visits. Since the datasets are de-identified, patients are not tracked over time. The 394,841 inpatient observations are probably not unique users, as it is likely that people with illicit drug SUD are admitted more than once. This may bias the results if groups who use certain illicit drugs frequent the ED and hospital more often. Additionally, there may be an implication for the statistical testing if the observations are not independent.

## Conclusion

Illicit drug use can devastate families. It places an extra burden on the police, court system, prisons, hospitals, EMS, and social workers, to name a few. Additionally, it affects everyone financially. Healthcare costs are high of patients with illicit drug SUD and poisoning, over half of which are paid for with tax payer dollars and to an unknown degree hospital write-offs. Injuries and illness of patients using cocaine and multiple drugs are associated with more expensive ED care and opioids and inhalants are associated with more expensive inpatient care. Prevention and treatment programs for people who use cocaine, inhalants, opioids, and multiple drugs could potentially pay for themselves in terms of healthcare costs averted. Particularly, the data provided shows how much Medicare and Medicaid is spending on healthcare costs of patients with illicit drug use. This research may help those who conduct cost benefit analyses or cost effectiveness analyses for substance use disorder programs.

Further research should include the total costs of healthcare associated with illicit drug use in ambulatory care. In addition, future research may help explain why cocaine inpatient healthcare costs more than doubled in 1 year but did not have the corresponding increase in counts.

## Data Availability

The datasets generated and/or analysed during the current study are available in the Agency for Healthcare Administration repository, [https://www.floridahealthfinder.gov/researchers/orderdata/order-data.aspx].

## Abbreviations

AHCA: Agency for Health Care Administration
ADHD: Attention deficit hyperactivity disorder
ED: Emergency department
ICD-10-CM: International Statistical Classification of Diseases and Related Health Problems Clinical Modification
LOS: Length of stay
NIDA: National Institute on Drug Abuse
ACA: Patient Protection and Affordable Care Act
PTSD: Post-traumatic stress disorder
SAMHSA: Substance Abuse and Mental Health Services Administration
SUD: Substance use disorder
